# Characterization of TMC-1 in *C. elegans* sodium chemotaxis and sodium conditioned aversion

**DOI:** 10.1186/s12863-020-00844-4

**Published:** 2020-03-30

**Authors:** Joseph Dao, Aileen Lee, Dana K. Drecksel, Nicole M. Bittlingmaier, Theodore M. Nelson

**Affiliations:** 1 Department of Human Science, Georgetown University Medical Center, Washington, DC 20057 USA; 2 Department of International Health, Georgetown University Medical Center, Washington, DC 20057 USA

**Keywords:** TMC-1, Chemotaxis, Sodium, Salty taste hedonics, Gustatory plasticity

## Abstract

**Background:**

While sodium is attractive at low and aversive at high concentrations in most studied species, including *Caenorhabditis elegans*, the molecular mechanisms behind transduction remain poorly understood. Additionally, past studies with *C. elegans* provide evidence that the nematode’s innate behavior can be altered by previous experiences. Here we investigated the molecular aspects of both innate and conditioned responses to salts. Transmembrane channel-like 1 (*tmc-1*) has been suggested to encode a sodium-sensitive channel required for sodium chemosensation in *C. elegans*, but its specific role remains unclear.

**Results:**

We report that TMC-1 is necessary for sodium attraction, but not aversion in the nematode. We show that TMC-1 contributes to the nematode’s lithium induced attraction behavior, but not potassium or magnesium attraction thus clarifying the specificity of the response. In addition, we show that sodium conditioned aversion is dependent on TMC-1 and disrupts not only sodium induced attraction, but also lithium.

**Conclusions:**

These findings represent the first time a role for TMC-1 has been described in sodium and lithium attraction in vivo, as well as in sodium conditioned aversion. Together this clarifies TMC-1’s importance in sodium hedonics and offer molecular insight into salt chemotaxis learning.

## Background

The principal mechanism for transduction of salty taste involves the passage of cations through specific ion channels within the apical membrane of receptor cells [1]. Pure salty taste is defined as the taste elicited by sodium chloride. In humans, while many non-sodium salts such as potassium have aspects of salty taste, their saltiness is accompanied by additional qualitative attributes most frequently described as bitterness [2]. In mammals, suggested components of the salty taste pathway include an amiloride-sensitive, cation selective (Na^+^ and Li^+^) epithelial sodium channel (ENaC) and an amiloride-insensitive, cation generalist transient receptor potential cation channel subfamily V family 1(TrpV1) [3, 4]. Moreover, salt is unique in that increasing concentration can induce a powerful aversion from the innate appetitive stimulus. Studies suggest that ENaC may be required for low-concentration salt taste; and the high-concentration salt taste pathway differentially recruits aversive taste pathways [3, 5, 6]. Some evidence suggests TrpV1 may also be involved in the high salt pathway [4]. Much remains unknown regarding the specificity of salt responses and the innate attractive and aversive properties of salts. Further understanding here can offer insights into how hedonics can contribute to the body’s mechanisms of balancing ions.

Analogous to humans, naive *C. elegans* display chemoattraction toward low concentrations of salt while avoiding high concentrations [7]. The chemoattraction to salts is thought to be mediated by four pairs of amphid sensory neurons ADF, ASE, ASG, and ASI [8]. Ablation of the ASE neurons results in a greatly reduced attraction response to sodium, with a residual response that is likely mediated by ADF, ASG, and ASI neurons. In contrast, avoidance of sodium was found to be mostly mediated by the ASH sensory neurons, which are primarily responsible for sensing chemical repellants and avoiding noxious stimuli such as high osmolarity and nose touch [9, 10]. In the absence of ASH, there is evidence that sensory neuron ADL can modulate sodium avoidance [10].

Recently, transmembrane channel-like 1 (*tmc-1*) in *C. elegans* has been suggested to encode a protein expressed in ASH that may be sodium-sensitive and/or alkali-activated with reports linking it to sodium and alkaline chemosensation [11, 12]. Transmembrane channel-like (TMC) genes encode a family of channel-like proteins that are evolutionarily conserved in humans and nematodes [13, 14]. In humans and mice, mutation of transmembrane channel-like 1, *TMC-1* is known to cause dominant and recessive forms of deafness [14–16]. While previous studies suggest that TMC-1 is a mechano-electrical transducer channel, there is evidence that it could function independently as an ion channel [16, 17]. Recent data suggests that TMC-1 in nematodes may mediate Na^+^-leak currents to stabilize membrane potential in excitable cells, including amphid sensory cells [18].

Utilizing transgenic lines expressing a fluorescent reporter under the *tmc-1* promoter, Chatzigeorgiou et al. observed that *tmc-1* is expressed primarily in aforementioned sensory neurons ASH, ADF, ASE, and ADL, in addition to PHA. By applying an escape “drop test” assay [9], Chatzigeorgiou et al. concluded that *tmc-1* mutants were strongly defective in the avoidance of high sodium concentrations above 100 mM, as mediated by activity in the ASH neuron. These authors additionally found that heterologous expression of TMC-1 in non-salt responsive ASK amphid neurons was sufficient to confer sodium sensitivity to these cells and finally showed that TMC-1 is sufficient to generate a sodium receptor in vitro. Together their data suggests that TMC-1 in the ASH neuron may function directly as a sodium receptor, leading to the aversive effects of high sodium.

However more recently, Wang et al. were unable to confirm a role for TMC-1 in sensing high concentrations of sodium or sodium chemosensory aversion in the nematode, even with similar methodologies. In contrast, they found that sodium sensation by the ASH neuron is dependent on the G protein ODR-3, opening the door for the possibility that high sodium aversion is mediated by a GPRC. Additionally, these authors concluded that TMC-1 is necessary for alkalinity sensation in the ASH neuron [12]. Ultimately, the channel’s role in sodium chemosensation remains in question.

Additional studies in *C. elegans* provide evidence that the nematode’s behavior can be altered by previous experiences. Most notably, in a phenomenon known as gustatory plasticity, prolonged pre-exposure to a variety of salts has been shown to induce aversion to innately appetitive low salt concentrations [19]. Pre-exposure to salts can abolish chemoattraction to compounds in a partly salt specific yet reversible manner. To measure the specificity of the conditioning, Jansen et al., utilized a cross-adaptation assay in which nematodes pre-exposed to NH_4_Cl were found to significantly reduce chemotaxis to NaOAc. However, the conditioned avoidance intensity was less than when nematodes were pre-exposed to NaOAc suggesting the presence of both a salt-specific and an aspecific response [19]. In a series of follow up studies it was hypothesized that gustatory plasticity following pre-exposure to NaCl leads to an ASE induced signal that sensitizes ADF, ADL, ASI, and ASH neurons ultimately resulting in aversion. G-protein signaling, serotonin, and glutamate have all been implicated in gustatory plasticity [20, 21]. A related phenomenon called salt chemotaxis learning has been shown to be dependent on insulin signaling and the calcium/calmodulin-dependent kinase, CMK-1 [22–25]. Here nematodes will avoid sodium following a period of starvation in the presence of NaCl. Much remains unknown regarding the mechanism of other signaling pathways and genes that may contribute to gustatory plasticity.

Here we sought to clarify the role of TMC-1 in the *C. elegans* salty taste pathway. In our study, we further characterized TMC-1 using a chemotaxis behavior assay developed by Wicks et al. to assess TMC-1’s contribution to chloride salts taste pathways for sodium, lithium, potassium, and magnesium [26]. Our findings suggest that while TMC-1 is required for NaCl and LiCl induced attraction behaviors, the channel likely has no significance in NaCl induced avoidance behaviors or the MgCl_2_ and KCl induced attraction behaviors. Additionally, we show that pre-exposure to NaCl not only disrupts NaCl induced attraction, but also LiCl. Further, conditioning to sodium is dependent on TMC-1. The findings of our project clarify TMC-1’s role in sodium chemotaxis in the nematode.

## Results

### TMC-1 contributes to *C. elegans*’ salty taste pathway

**Fig. 1:**
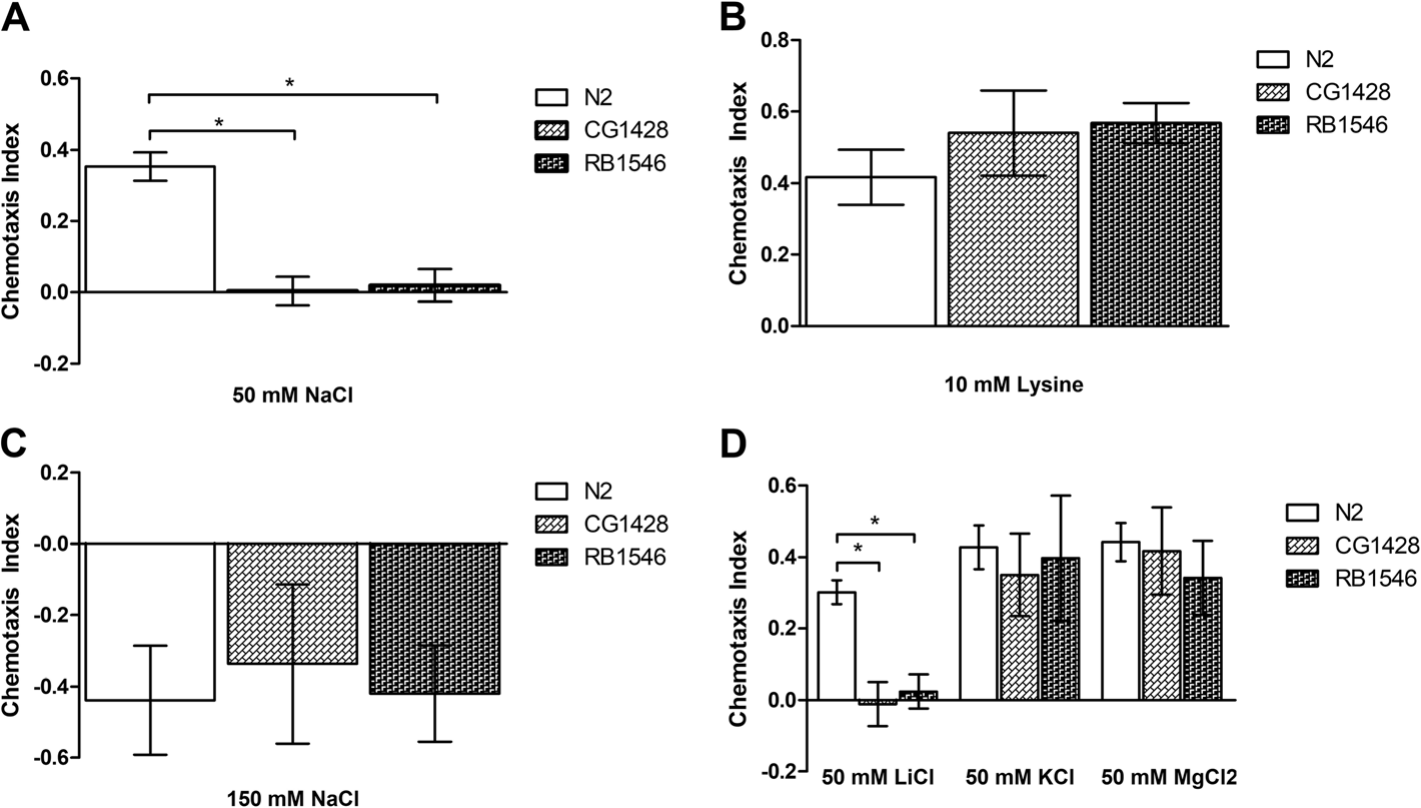
TMC-1 contributes to *C. elegans*’ salty taste pathway. Each individual chemotaxis assay contained at least 100 nematodes, data reported here as average of independent assays (n). Each data point represents a minimum of three assays. Chemotaxis index (CI) has a range of − 1 to 1, where positives values indicate attraction to tastant, negative values indicate aversion, and values close to 0 indicate no preference. Error bars represent SEM. Asterisks identify significant strain effects as indicated by planned LSD tests following one-way ANOVA (*p* < 0.05). **(a).** TMC-1 is required for sodium induced attraction behaviors. *Tmc-1* mutants CG1428 and RB1546 behaved significantly different from wild-type N2 toward 50 mM NaCl (n: N2 = 6, CG = 5, RB = 4). **(b).** Mutation of *tmc-1* does not disrupt the lysine taste pathway (n: N2 = 5, CG = 5, RB = 4). *Tmc-1* mutants exhibited no statistically significant difference from wild-type in their attraction to 10 mM lysine. **(c)**. TMC-1 is not required for sodium-induced avoidance behaviors. (n: N2 = 4, CG = 4, RB = 5) **(d).** TMC-1 contributes to non-sodium salt induced attraction behaviors. Mutation of *tmc-1* disrupts the nematodes’ attraction to 50 mM LiCl (n: N2 = 4, CG = 4, RB = 4), however there was no statistically significant difference in the nematodes’ behavior toward 50 mM KCl (n: N2 = 7, CG = 10, RB = 7) or 50 mM MgCl_2_ (n: N2 = 8, CG = 3, RB = 5)

*C. elegans* are innately attracted to sodium at low concentrations [7]. To address whether TMC-1 contributes to the *C. elegans* chemoattraction to sodium, we first performed chemotaxis behavior assays with increasing concentrations of NaCl (pilot data not shown). We observed that the wild-type nematodes (N2) most preferentially migrated toward 50 mM NaCl (Fig. 1a). Notably, we found that the *tmc-1* mutant nematodes (RB1546 and CG1428) were defective in their attraction behavior (One-way ANOVA, *F*_2,12_ = 29.2, *P* < 0.001). To ensure that we did not disrupt the nematode’s attraction behavior to all tastants, we used 10 mM of lysine, an amino acid as a positive control (Fig. 1b). In contrast to 50 mM NaCl, mutation of *tmc-1* did not disrupt the nematode’s attraction behavior toward the lysine quadrants (One-way ANOVA, *F*_2,9_ = 1.5, *P* = 0.273). These results suggest that TMC-1 is required for sensing low concentrations of sodium and sodium induced attraction behaviors.

When nematodes encounter noxious chemical repellents, they immediately reverse and turn to change their direction of movement. *C. elegans* are known to avoid high concentrations of sodium [27]. Previous studies found TMC-1 expressed in the ASH neurons, which are primarily responsible for sensing chemical repellants and avoiding noxious stimuli; however, there has been a discrepancy on whether TMC-1 contributes to sodium avoidance [11, 12]. To address whether mutation of *tmc-1* could also disrupt sodium avoidance, we performed chemotaxis behavior assays with 150 mM NaCl, which we observed the wild-type nematodes strongly avoided (Fig. 1c). Subsequently, we found that the *tmc-1* mutant nematodes showed no notable differences to the wild-type suggesting that their ability to avoid sodium remained intact (One-way ANOVA, *F*_2,10_ = 0.096, *P* = 0.909). These results support that TMC-1 does not have a major role in sensing high concentrations of sodium and is not required for sodium induced avoidance behaviors.

Other than sodium, *C. elegans* have the ability to detect other cations such as lithium, potassium, and magnesium [7]. Specificity studies using calcium imaging and behavior assays found that mutation of *tmc-1* did not affect the nematode’s behavior toward magnesium, calcium, or potassium suggesting that the channel could be sodium specific [11]. To test TMC-1’s specificity level ourselves in vivo, we chose to measure the nematodes’ behavior toward lithium, potassium, and magnesium chloride salts (Fig. 1d). We observed that the wild-type nematodes exhibited attraction behaviors toward 50 mM LiCl, KCl, and MgCl_2_ agar. In line with previous findings, we observed no notable differences between the *tmc-1* mutants and the wild-type toward KCl (One-way ANOVA, *F*_2,21_ = 0.101, *P* = 0.905) and MgCl_2_ (One-way ANOVA, *F*_2,13_ = 0.436, *P* = 0.656); however, we found that the *tmc-1* mutants were defective in their ability to migrate to the LiCl quadrants (One-way ANOVA, *F*_2,9_ = 12.3, *P* = 0.003). These results suggest that while TMC-1 is required for lithium induced attraction behaviors, the channel does not contribute to magnesium or potassium induced attraction behaviors.

### Effects of sodium conditioning on salty taste pathways

**Fig. 2:**
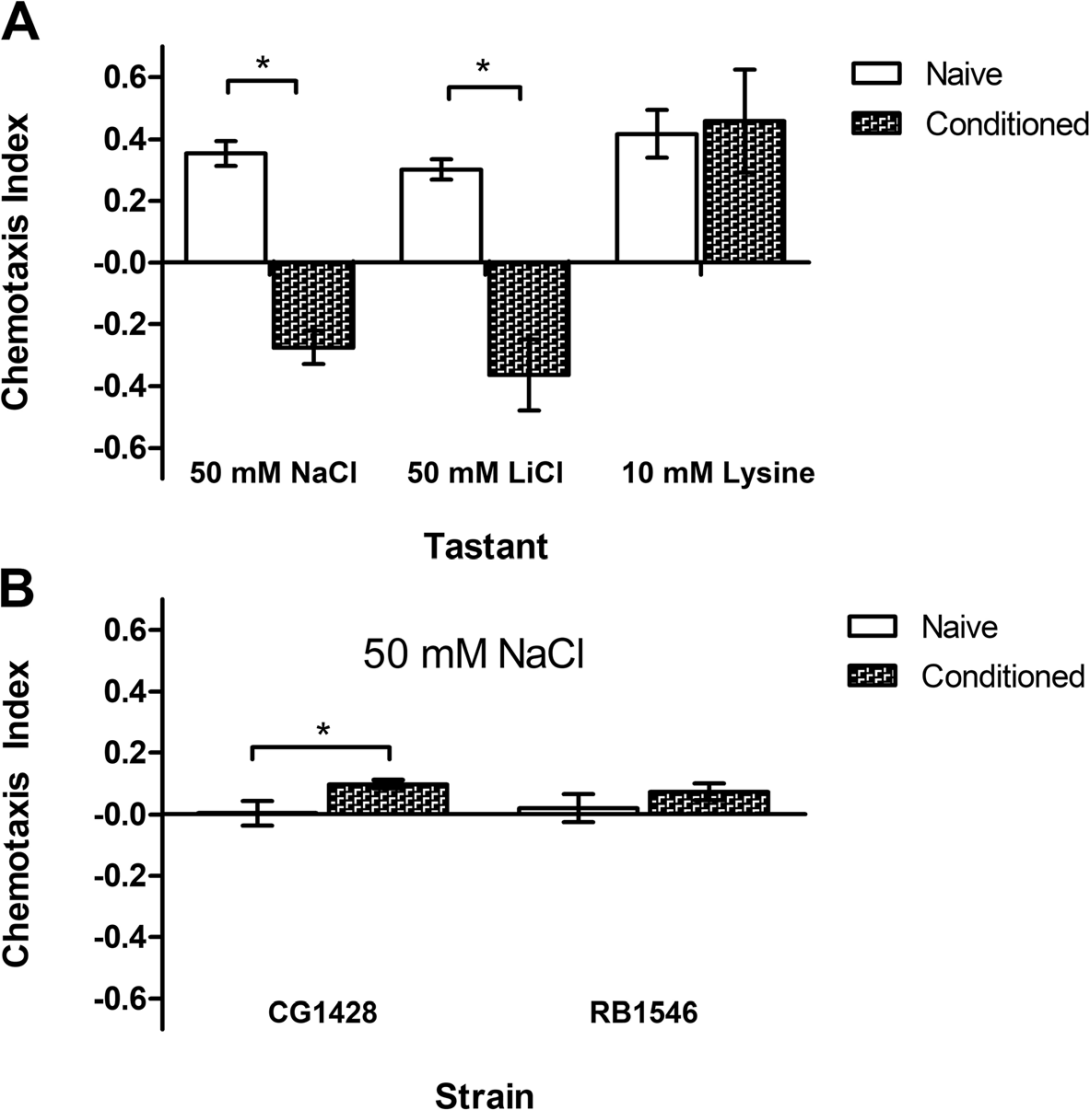
The effects of sodium conditioning on chemotaxis. Each individual chemotaxis assay contained at least 100 nematodes, data reported here as average of independent assays (*n*). Each data point represents a minimum of three assays. Nematodes were conditioned for 20 min in either CTX buffer or CTX buffer containing 50 mM NaCl. Chemotaxis index (C.I) has a range of − 1 to 1, where positives values indicate attraction to tastant, negative values indicate aversion, and values close to 0 indicate no preference. Error bars represent SEM. **(a)** Sodium conditioning disrupts wild-type nematodes’ NaCl and LiCl attraction pathway. Sodium conditioned N2 nematodes behaved significantly different from their naïve counterpart toward 50 mM NaCl (n: naïve = 6, conditioned = 5) and 50 mM LiCl (n: naïve = 4, conditioned = 4). There was no statistically significant difference in their behavior toward 10 mM lysine (n: naïve = 5, conditioned = 5). For this and panel B, asterisks identify significant conditioning effects as indicated by unpaired Student’s t-test (*p* < 0.05). **(b).***Tmc-1* mutation disrupts nematodes’ innate attraction and induced aversion to 50 mM NaCl. (n: CGnaïve = 5, CGconditioned = 5, RBnaive = 4, RBcondtioned = 4)

Previous studies have indicated aversion to innately appetitive low salt concentrations could be induced after pre-exposure to salt in *C. elegans.*^19^ To investigate the specificity of this phenomenon, we chose to measure the nematode’s behavior toward 50 mM sodium and lithium chloride salts following the sodium conditioning. In line with previous work, we observed that conditioning disrupted the nematode’s innate attractive behavior toward 50 mM NaCl (unpaired Student’s t-test, *P* < 0.001) (Fig. 2a). Similarly, the sodium conditioning disrupted the nematode’s attraction to LiCl (unpaired Student’s t-test, *P* < 0.001) but not lysine (unpaired Student’s t-test, *P* = 0.825) (Fig. 2a). Our findings indicate that sodium conditioning contains an aspecific effect that disrupts attraction to non-sodium (lithium) salts.

Given that our previous experiments showed that TMC-1 was not required for sodium aversion, we sought to investigate if TMC-1 was required for the nematodes salt chemotaxis learning. Specifically, we wanted to determine if the *tmc-1* mutants were defective in their ability to become aversive to innate attractive sodium concentrations following the sodium conditioning via gustatory plasticity. To address this, we performed chemotaxis behavior assays with the nematodes at 50 mM NaCl. We observed that *tmc-1* mutants exhibited no attraction or aversion post-conditioning. (Fig. 2b). While CG1428 nematodes’ behavior was statistically significantly different between naïve and conditioned, the trends reflect very little attraction and aversion (unpaired Student’s t-test, *P* = 0.01). No difference in behavior was observed for RB1546 (unpaired Student’s T-test, *P* = 0.42). Overall this suggests an ability to detect sodium through TMC-1 is required for conditioned aversion.

## Discussion

The major goal of this study was to further investigate how mutation of *tmc-1* and/or pre-exposure to sodium influences *C. elegans’* salty taste pathway and behavior. *Tmc-1* has been suggested to encode a sodium sensitive channel in the nematodes, but its role in salty taste still remains unclear. Here we compared the behavioral responses of wild-type nematodes (N2) to two different strains of *tmc-1* mutants; RB1546 (a deletion) and CG1428 (a missense mutation). Previous studies investigating the role of TMC-1 in sodium sensing have utilized only the RB1546 strain [11, 12]. To the best of our knowledge this is the first-time sodium sensing behavior has been reported for the CG1428 strain. In all cases the two mutant strains behaved similarly. Recently, there has been a discrepancy on whether TMC-1 has a major role in sensing high concentrations of sodium [11, 12]. In this study, we conclude that TMC-1 is not required for sodium induced avoidance behavior and as such does not have a major role in sensing high concentrations of sodium. For our high concentration of sodium, we used 150 mM NaCl as it is isosmotic to the nematode and we could avoid any adverse effects of osmolarity. This concentration is consistent with previous chemotaxis investigations that classify NaCl concentrations above 100 mM as high [26]. Our results suggest that high concentrations of sodium may be sensed by an alternative sodium sensitive channel. The mechanisms mediating sodium aversion in both nematodes and humans remain unidentified, leaving open the possibility that they share commonalities. Further investigation is needed to confirm and address this gap.

Our results regarding TMC-1 and sodium aversion do not align with the conclusion of Chatzigeorgiou et al. that *tmc-1* mutants are strongly defective in the avoidance of high sodium concentrations [9]. Several important differences in methodology should be noted. Chatzigeorgiou et al. utilized a drop test assay in which cultured nematodes were assayed by applying a drop of stimulus near the tail region and direction of movement was used to indicate avoidance or non-avoidance. We used a chemotaxis behavior assay developed by Wicks et al. [27] In our assays (see methods), nematodes were allowed access to agar pads both imbedded with stimulus and without. Our measure of avoidance was the proportion of nematodes on non-stimulus agar vs. on stimulus-containing agar post 20 min. Additionally, we age synchronized our nematodes with a bleaching assay followed by an overnight incubation of L1 nematodes in a sodium containing buffer without bacterial food source. It is possible that the two different assays (brief stimulus presentation vs. prolonged stimulus presentation) are activating separate avoidance pathways. Further, we cannot rule out the that the early life exposure to sodium impacts the behavioral responses we see in our adult nematodes, similar to the phenomenon of olfactory imprinting [28]. Still, in our study both *tmc-1* mutants and wild-type controls underwent the same procedures, and there was no observable defect in sodium aversion. Further, it should be noted that when using the same methodologies as Chatzigeorgiou et al., Wang et al. were unable to confirm a role for TMC-1 in sensing high concentrations of sodium [12].

While we did not find evidence that TMC-1 contributes to sodium induced avoidance behaviors, we did observe that *tmc-1* mutants were defective in sodium induced attraction behaviors suggesting the TMC-1 is critical for sensing low concentrations of sodium (Fig. 1a). Moreover, we found that the *tmc-1* mutants were defective in lithium induced attraction behaviors. However, in line with previous studies, we also observed that *tmc-1* mutants retained their potassium and magnesium induced attraction behaviors suggesting that TMC-1 is neither sodium specific nor a cation generalist. To our knowledge, this is the first time a role for TMC-1 in sodium attraction and lithium sensation has been described in vivo.

To investigate how pre-exposure to sodium affected the nematodes’ salty taste pathway and behavior, we conditioned the nematodes in CTX buffer containing 50 mM NaCl before subjecting them to chemotaxis behavior assays. We concluded that the sodium conditioning, in line with previous studies [19], disrupts nematodes’ attraction to 50 mM NaCl but also 50 mM LiCl. While the mechanism underlying this phenomenon still remains unclear, one possible explanation for this is that sodium conditioning elicits both a salt specific and an aspecific response [19]. As such, we believe that due to overlapping attractive pathway, likely TMC-1 mediated, nematodes were able to generalize the conditioned response to sodium onto lithium.

Our chemotaxis behavior assays with sodium conditioned *tmc-1* mutants showed no discernable behavioral differences to their naïve counterpart. The behavioral data that we present here does not allow us to conclusively describe mechanism. However, several lines of evidence suggest that *tmc-1* mutation is having a sodium specific effect and is not more generally altering signally or motor pathways. First, *tmc-1* mutants show wild-type levels of attraction to lysine, potassium, and magnesium. Second, *tmc-1* mutants show normal aversion to high concentrations of sodium. One plausible explanation for the observed conditioning differences in *tmc-1* mutants is that for the nematodes to learn to avoid sodium, they must be able to taste or sense the sodium in the Na-CTX buffer. As such, we believe the sodium concentration in the buffer we utilized in our investigation was likely below detection threshold for the mutant nematodes in the absence of TMC-1. It could be hypothesized that the ASE neuron, in the absence of TMC-1, is unable to generate the sodium-dependent signals necessary to sensitize ADF, ADL, ASI, and ASH neurons and thereby induce aversion. Future chemotaxis behavior assays using nematodes conditioned in higher concentrations of sodium such as 100 mM or 150 mM could strengthen this claim. Selective rescue of TMC-1 in ASE neurons and calcium imaging of ASE in mutant nematodes would also support this.

## Conclusions

Here we further investigated TMC-1 and sodium conditioning and clarified the contribution of both to salty taste in *C. elegans.* Our results suggest that TMC-1 contributes to sodium and lithium induced attraction behavior, but not potassium or magnesium, suggesting the response is neither entirely sodium specific or generalized for cations. We show that sodium conditioning disrupts not only sodium induced attraction, but also lithium. Lastly, we found that TMC-1 is required for gustatory plasticity following pre-exposure to 50 mM NaCl.

## Methods

### Nematode strains and maintenance

Three strains of *C. elegans*: wild-type N2 and two *tmc-1* mutants RB1546 (2025 bp homozygous deletion of *tmc-1*) and CG1428 (L376I) were used in this project. The N2 strain was a gift from the laboratory of Andy Golden. RB1546 and CG1428 strains were provided by the *C. elegans* Gene Knockout Project at the Oklahoma Medical Research Foundation as part of the International *C. elegans* Gene Knockout Consortium. Nematodes were cultured as previously described [29] on 60 mm petri dishes of Modified Youngren’s, Only Bacto-peptone, MYOB [30] (2% Bacto Agar (BD 214050), 34 mM NaCl, 3.5 mM Trizma® HCl, 2 mM Trizma® Base, 0.31% Bacto Peptone (BD 211677), and 0.02 mM cholesterol) and stored at 21 °C. 80 μL of *E. coli* OP50 bacteria in 2.5% Difco™ LB broth (BD 244620) was pre-seeded onto the plates to serve as the nematode’s food source. Strains were maintained by transferring the nematodes from populated plates onto fresh MYOB plates pre-seeded with OP50 every three days.

### Synchronization of nematode growth via bleaching

To ensure that our data reflected the behavior of the nematodes at a uniformed age, a 20% alkaline hypochlorite solution (20% bleach and 0.25 M NaOH) was utilized to synchronize the nematode growth cycle prior to each behavior assay [31].

Briefly, 5 mL of M9 buffer (22 mM KH 2 PO4, 42 mM Na2HPO4, 86 mM NaCl, and 1 mM MgSO4) was added to well-populated agar plates to detach the nematodes and eggs. Nematodes and eggs were collected via centrifugation (3.3 k x g for 90- s), M9 was discarded, and 7.5 mL of 20% alkaline hypochlorite solution added. Nematodes were incubated with inversion for two and half minutes in the hypochlorite solution. The solution works to preserve eggs while degrading adult and larval-stage nematodes. Eggs were again collected via centrifugation, the hypochlorite solution discarded, and an M9 wash added. The eggs were washed twice more in M9 and left overnight in M9 at room temperature. The following day, now L1, nematodes were transferred to MYOB plates pre-seeded with OP50. Nematodes were incubated at 21 °C for 50–60 h, allowing them to mature into an adult stage cohort.

### Conditioning and Chemotaxis behavior assay

To assess the nematodes’ behaviors and preference to salt, a chemotaxis behavior assay was performed [19–21, 27]. Adult nematodes were conditioned in either 5 mL of CTX buffer (5 mM KH2PO4/K2HPO4 at pH 6, 1 mM CaCl2*2H2O, and 1 mM MgSO4*7H2O) or CTX buffer containing 50 mM NaCl (Na-CTX) for 20 min, allowing adequate exposure time. The nematodes were then transferred immediately onto the center of an agar filled assay plate with alternating quadrants of non-taste agar (2% Bacto Agar (BD 214050), 5 mM KH2PO4/K2HPO4 pH 6, and 5 mM MgSO4) and taste agar containing 50 mM NaCl, 150 mM NaCl, 50 mM LiCl, 50 mM MgCl_2_, or 10 mM Lysine). A non-tastant agar bridge was placed over the plastic dividers of the assay plates to ensure that the nematodes could migrate between quadrants. Nematodes were allowed to chemotax on the plate for twenty minutes before each quadrant was counted and a prevalence was noted.

A Chemotaxis Index (CI) was calculated to quantify the nematodes’ response to the tastant and non-tastant environments. This was determined by calculating the proportion of the nematodes on the given agars with the formula: *CI = (T – N) / (T + N)*, where T is the number of nematodes on the two tastant quadrants and N is the number of nematodes on the two non-tastant quadrants. Therefore, a positive CI value indicates attraction toward the tastant and a negative CI value indicates aversion.

Statistical analysis by one-way ANOVA, followed by a least significant difference (LSD) post hoc test was performed using IBM® SPSS® Statistic 25. Standard error of mean (SEM) was calculated and Student’s unpaired t-test was performed using GraphPad Prism 5.

## Data Availability

The datasets used and/or analyzed during the current study are available from the corresponding author on reasonable request.
